# Serum Selenium Level in Patients with Gastric Non-Cardia Cancer and Functional Dyspepsia

**Published:** 2015-05

**Authors:** Zahra Hosseini Nezhad, Sodaif Darvish Moghaddam, Mohammad Javad Zahedi, Mehdi Hayatbakhsh, Fariba Sharififar, Farzane Ebrahimi Meimand, Mahdieh Nazari

**Affiliations:** 1 Department of Biochemistry, Medical School, Kerman University of Medical Science, Kerman, Iran;; 2 Physiology Research Center, Department of Internal Medicine, Afzalipour Hospital, Kerman University of Medical Science, Kerman, Iran;; 3 Herbal and Traditional Medicines Research Center, Faculty of Pharmacy, Kerman University of Medical Science, Kerman, Iran;; 4 Department of Cardiology, Kerman University of Medical Sciences, Kerman, Iran

**Keywords:** Selenium, Gastric cancer, Helicobacter pylori

## Abstract

**Background:**

Gastric cancer (GC) is the most common gastrointestinal cancer in Iran. Helicobacter pylori (*H. pylori*) accounts as one of the main risk factors for gastric non-cardia cancer (GNCC). It is suggested that high serum selenium level may have a protective role in GNCC. In this cross-sectional study, we determined the serum Se level and the status of *H. pylori *infection in two populations with GC and functional dyspepsia (FD).

**Methods:**

The enrolled patients were 85 (27 women, 58 men) with recent pathologically proven GNCC (adenocarcinoma) and 85 (34 women, 51 men) FD patients. Serum Se was measured by atomic absorption spectrophotometry. *H. pylori* IgG antibody was detected by quantitative enzyme immunoassay.

**Results:**

The mean age in the GNCC and FD patients were 62.85±14.6 and 58.9±14.7 years, respectively (P=0.08). The serum selenium levels were 111.6±27.7 and 129.9±32.1 μg/L (mean±SD) in GNCC and FD patients, respectively (P<0.001). The frequency of *H. pylori* infection was 49.4% (n=42) and 68.2% (n=58) in GNCC and FD patients (P=0.013). The crude and adjusted odds ratio (OR) between GNCC and the linear effect of serum selenium level were 0.98 and 0.982, respectively (P=0.002). This means that each unit increase in serum selenium level decreases the odds of cancer by 2%.

**Conclusion:**

Serum selenium level was significantly lower in GNCC cases. It suggests that lower serum selenium might have some association with the risk of GNCC. *H. pylori* infection does not play a significant impact on this association.

## Introduction

Selenium (Se) is from the group of VI elements which has been studied for antioxidant and anticancer properties,^1-3^ especially against gastric cancer (GC).^4^^,^^5^ Oxidative stress can induce carcinogenic process. Se exists in the human body in the form of selenoproteins and selenocysteines. The important metabolic functions of Se are supposed to be due to the protection of membrane lipids and macromolecules from oxidative damage by combating against reactive oxygen species; and the activation of antioxidant proteins including glutathione peroxidase, thioredoxin reductase, leading to decreased levels of hydrogen peroxide. Se also regulates the G1-phase of the cell cycle, DNA damage, and controls cell mediated immunity and B-cell function.^6^^,^^7^

Although ecological and animal studies have suggested that Se is involved in the reducing the risk of cancer, there are many controversial studies regarding the protective/therapeutic role of Se in human cancer. In addition, high Se level may have adverse effects on carcinogenesis of gastric non-cardia cancer (GNCC).^8^ On the other hand, an inverse association between serum Se level and gastric cancer risk was observed in countries with low gastric cancer risk such as Finland and the Netherlands.^9-11^ In a study by Charalabopoulos et al. on gastric cancer cases, they concluded that decreasing levels of serum Se might be involved in the development and progression of gastric carcinoma.^12^

Despite recent decline, gastric cancer is the fourth most common cancer and the second leading cause of cancer-related death worldwide. Two main sites of gastric adenocarcinoma are proximal (cardia) and distal (noncardia) regions.^13^ However, the incidence of gastric cardia cancer (GCC) increased during the last decades,^14-16^ but GNCC still remains the major health problem, especially in different parts of Iran.^15-17^

Esophageal and gastric cancers are the two most common causes of cancer death in Iran. Recent cancer registry data showed highly varying rates of these cancers in four provinces of Iran, namely Ardabil, Mazandaran, Golestan, and Kerman.^4^

In a recent published review (2010), gastric cancer still stands as the first prevalent cancer in Iranian men and the third most common cancer in Iranian women.^18^

According to studies during the last decade in Kerman province (southeast of Iran), overall, the gastric cancer was the third prevalent cancer with a prevalence of 9.38 in 100,000 individuals. Among the gastrointestinal (GI) cancers, it ranked as the most prevalent, followed by esophageal cancer.^19^ In another study, Kerman was known as the fourth province for gastric cancer prevalence, placed after Ardabil, Semnan and Golestan, with an average of 5.1 for women and 10.2 for men per 100,000.^15^

*H. pylori* accounts as the main known risk factor for GC.^20^ Its prevalence in Iran has been reported to be between 27% and 89%^21^ with 61.6% in Kerman province.^22^ Despite the high prevalence of *H. pylori* infection, the variations of intra-country GC prevalence cannot be explained solely by *H. pylori* infection. Thus, the role of other environmental factors needs to be investigated. As the protective effect of serum Se in GC has been debated,^8^^,^^23^ it seems necessary to scrutinize the effect of both Se deficiency and *H. pylori* infection in gastric cancer occurrence. This study was conducted to determine the serum Se level and *H. pylori* status in GNCC patients compared with functional dyspepsia (FD) individuals.

## Patients and Methods

This case-control study was carried out on 170 patients (109 men and 61 women). Eighty-five patients with recent pathologically proven GNCC (as the cases) were compared with 85 FD individuals (age/sex matched as the controls). The eligible participants who referred to the Afzalipour Hospital GI clinic were enrolled in the study. Cases of gastric cancer were identified and confirmed by upper GI endoscopy and histology. Control group cases were selected from those who had referred with minor upper abdominal symptoms. After a thorough physical examination, routine lab tests, upper GI endoscopy, and abdominal ultrasound were performed. Those patients without abnormal findings were labeled as functional dyspepsia and they were enrolled in the study. The reasoning for the selection of FD patients as the control group was to do investigations ethically and to accurately determine their health status. Beside of GNCC in cases, those patients with a major health problem (e.g. diabetes mellitus, chronic kidney disease, chronic liver disease, coronary artery disease, underlying malignancy, prolonged fever, taking many medications) were excluded from the study in both groups.

7 mL of fasting blood sample was obtained from all participants. The blood was drawn before any therapeutic intervention, including surgery, radiotherapy, or chemotherapy in the case group. *H. pylori* infection was identified by measuring serum IgG by enzyme immunoassay method (Monobind Inc., USA). The preserved sera (stored at -20^º^C) were used for Se level measurement by atomic absorption spectrophotometry (VA-220A, Varian, Australia). Informed consent was obtained from all participants.

*Statistical Analysis*

SPSS software version 15 (SPSS Inc., Chicago, IL, USA) was used for statistical analysis. The descriptive data were shown by the frequency and mean±SD. The data were analyzed by Chi-square test, *t* test, and logistic regression test. P<0.05 was considered statistically significant.

## Results

A total of 170 patients were enrolled in the study. In GNCC, 58 (68.2%) and 27 (31.8%) cases were male and female, respectively. In FD group, there were 51 (60.0%) men and 34 (40.0%) women (P=0.337). The mean age of the GNCC and FD patients were 62.85±14.6 and 58.9±14.7 years, respectively (P=0.08).

Serum Se level was 111.6±27.7 µg/L in gastric cancer patients and it was 129.9±32.1 µg/L in FD patients (P<0.001) (table 1).

**Table 1:** Serum Se level in gastric non-cardia cancer and functional dyspepsia patients

**Groups**	**Number **	**Mean serum Se (µg/L)**	**P value**
Cancer	85	111.6±27.7	<0.001
Non-cancer	85	129.9±32.1	

**Figure 1:**
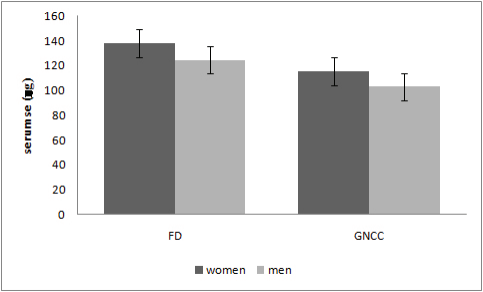
Mean serum Se values in GNCC and FD patients according to sex (P>0.05).

Overall, the serum Se level was higher in women than in men (127.8±31.2 vs. 116.8±30.8 µg/L, respectively) (P=0.028). Although the serum Se level was also higher in women, regardless of the case or the control group, it was not statistically significant. In this way, in GNCC, it was 115.2±25.5 µg/L for women and 102.9±28.7 µg/L for men (P=0.406); while in FD it was 137.8±32.0 µg/L for women and 124.6±31.4 µg/L for men (P=0.062) (figure 1).

Seroprevalence of *H. pylori* infection was positive in 42 (49.4%) cases of GNCC and in 58 (68.2%) patients of the FD group (P=0.028). Serum Se level according to *H. pylori* status in GNCC was: 114.7±29.0 µg/L for *H. pylori* positive and 108.6±26.4 µg/L for *H. pylori* negative patients (P=0.299) (table 2). By logistic regression analysis, the crude and adjusted odds ratio (OR) between GNCC and the linear effect of serum selenium level were 0.98 and 0.982, respectively (P=0.002). It means that each unit increase in serum selenium level decreases the odds of cancer by 2%.

**Table 2:** Serum selenium level in GNCC according to* H. pylori *status

** Positive *H. pylori***		** Negative *H. pylori***		
**No (%)**	**Se (µg/L)**	**No (%)**	**Se (µg/L)**	**P value**
42 (49.4)	114.71±28.99	43 (50.6)	108.56±26.39	0.299

## Discussion

A growing body of evidence has shown that Se may have anti-carcinogenic effects, especially against cancers of the lung, prostate, skin, and gastrointestinal system.^6^^,^^7^^,^^24^^,^^25^

In the present study, the serum Se level was significantly lower in GNCC cases compared with the FD patients. These results were comparable with the study of Ujiie,^5^ who reported significantly lower serum Se level in patients with any type of cancer in comparison with non-cancer cases, except in breast cancer. In this study, the average serum Se in healthy adults was 110.5 ppb, while it was 95.8 and 106.6 ppb in cancer and non-cancer patients. Thus, the low Se status should be considered as a risk factor for cancer, even in Japan; where Se intake is sufficient in healthy population.^5^ Previously, Nouraie et al. have shown differences in the average of serum Se in different provinces of Iran.^4^ The average serum Se in Ardabil, Kerman, Mazandaran and Golestan were 82, 119, 123, and 155µg/L, respectively. They suggested that the high incidence of gastric cancer and pre-neoplastic gastric lesions in Ardabil province could be partly due to low level of Se.^4^ In Koriyama et al. study, the Se level was high in both gastric cancer and non-cancer patients. They indicated that the inverse association between Se level and gastric cancer may occur only among populations with low Se levels.^8^

Some investigations reported prophylactic effects of Se supplementation on carcinogenesis; especially in populations where average dietary Se levels are low, but the optimum dietary Se intake is debated.^26^ On the other hand, administration of excessive doses of Se may also inhibit cellular proliferation.^3^

In our study, the prevalence of *H. pylori* in non-cancer individuals (68.2%) was higher than in gastric cancer patients (49.4%). It could be explained by selecting the control group from dyspeptic patients, even with minor digestive symptoms. In this regard, subgroup analysis was performed and no significant difference was observed between the Se level and *H. pylori *status in GNCC cases (P=0.299). The odds ratio (OR) between the linear effect of serum selenium and GNCC was 0.98 (P=0.002). These imply that despite the important role of *H. pylori* infection in gastric cancer, low Se level may predispose the patients to GNCC independently from *H. pylori* status.

In our study, the serum Se level was higher in all women than in all men (P=0.028), but it showed no significant difference between GNCC (P=0.406) and the FD (P=0.062) groups. In Ujiie’s study, the Se level was higher in men compared with women in both cancer and non-cancer patients.^5^ In an ecologic study on healthy adults in Iran, serum selenium concentrations did not differ in males (124 μg/L) and females (116 μg/L) (P=0.49).^4^

## Conclusion

There is a general understanding of the anticancer and antioxidant properties of Se. According to the results of some studies, including the present one, there are some evidences in favor of a relation between low Se level and GC, however this relation is not comprehensively established yet. GC is a multifactorial disease and the role of other factors should be considered. Before any recommendation, the relation between low Se and GC should be confirmed by more ecologic and clinical trial studies. In addition, food sources, bioavailability, and toxicity aspects of Se should also be in mind. 
